# Rare Presentation of Primary Classical Hodgkin Lymphoma of a Rectosigmoid Colon in a 79-Year-Old With a History of Irritable Bowel Syndrome

**DOI:** 10.7759/cureus.43465

**Published:** 2023-08-14

**Authors:** Mobeena Arif, Joseph Radakovitz, Pallavi Kulkarni, Iryna Hepburn

**Affiliations:** 1 Family Medicine Residency Program, WellSpan Good Samaritan Hospital, Lebanon, USA; 2 Family Medicine, Philadelphia College of Osteopathic Medicine, Philadelphia, USA; 3 Family and Community Medicine, Penn State University College of Medicine, Milton S. Hershey Medical Center, Hershey, USA; 4 Gastroenterology, Wellspan Good Samaritan Hospital, Lebanon, USA

**Keywords:** hodgkin lymphoma, primary lymphoma, classic hodgkin lymphoma, extra nodal non hodgkin lymphoma, irritable bowel disease, sigmoid colon

## Abstract

We discuss the case of a 79-year-old immunocompetent male who presented with weight loss and diarrhea and ultimately was found to have a rectosigmoid mass on a colonoscopy. Even though initial biopsies obtained during colonoscopy were non-diagnostic, considering the likelihood of malignancy, lower anterior resection was performed, and pathology confirmed the diagnosis of primary Hodgkin’s lymphoma of the sigmoid colon. Hodgkin’s lymphoma typically presents as painless supra-diaphragmatic lymphadenopathy with B symptoms such as fever, unexplained weight loss, and drenching night sweats. Due to the rarity of primary Hodgkin lymphoma in the colon and its non-specific initial presentation, we believe sharing this case will bring awareness to the atypical presentation of Hodgkin lymphomas.

## Introduction

Hodgkin lymphomas are lymphoid neoplasms that generally progress slowly. Forty percent (40%) of patients present with asymptomatic lymphadenopathy, a mass on chest X-ray, or constitutional symptoms [1]. The most commonly affected lymph nodes demonstrate Reed- Sternberg (R-S) cells mixed with non-neoplastic inflammatory cellular background on histology. In 60% to 80% of cases, cervical and supraclavicular nodes are involved [1]. Atypical presentations of lymphomas include extranodal sites, including the gastrointestinal tract, accounting for 40%, out of which 4% comprise non-Hodgkin lymphomas [1,2]. Therefore, primary lymphomas of the GI tract are rare and only account for 0.2-1.2% of all GI malignancies [1,2], predominantly confined to the small intestine, specifically the ileum. Only a few cases of primary GI lymphomas have been reported in the literature [1-4]. Herein, we present the case of a 79-year-old immunocompetent male with a diagnosis of primary classical Hodgkin lymphoma in the rectosigmoid area of the colon.

## Case presentation

A 79-year-old Caucasian male with a past medical history of irritable bowel syndrome (IBS) with diarrhea presented to his primary care physician (PCP) following a hip fracture with concerns for a 15-pound weight loss, decreased appetite, watery diarrhea, abdominal pain, and abdominal distention over a six-month period. The patient initially attributed these symptoms to his IBS and was started on Bentyl. Upon follow-up, the patient was noted to have lost additional 5 pounds over a two-month period with no symptomatic improvement. Stool testing for Clostridioides (C.) difficile, Salmonella, Shigella, Escherichia (E.) coli, Yersinia, and Campylobacter was negative. CT of the abdomen and pelvis was significant for cholelithiasis, rectal wall thickening, sigmoid diverticula, and multiple nonenlarged lymph nodes in the fat adjacent to the rectum. Due to abnormal CT findings, the patient was referred to a gastroenterologist for further evaluation. Laboratory findings five months prior to the patient's visit with the gastroenterologist are detailed in Table 1. Celiac disease panel, fecal fat, giardia antigen, and ova and parasite studies were unremarkable.

**Table 1:** Hematologic findings at numerous points in the case GI: gastroenterology; MCV: mean corpuscular volume

	Five months prior to the first visit with GI	Prior to biopsy	Post biopsy	10-month follow-up
Hemoglobin (g/dL)	9.3	11.1	9.3	12.9
Hematocrit (%)	28.7	34.5	28.5	39.3
MCV (fL)	100.7	96.9	95.6	98.7

**Figure 1:**
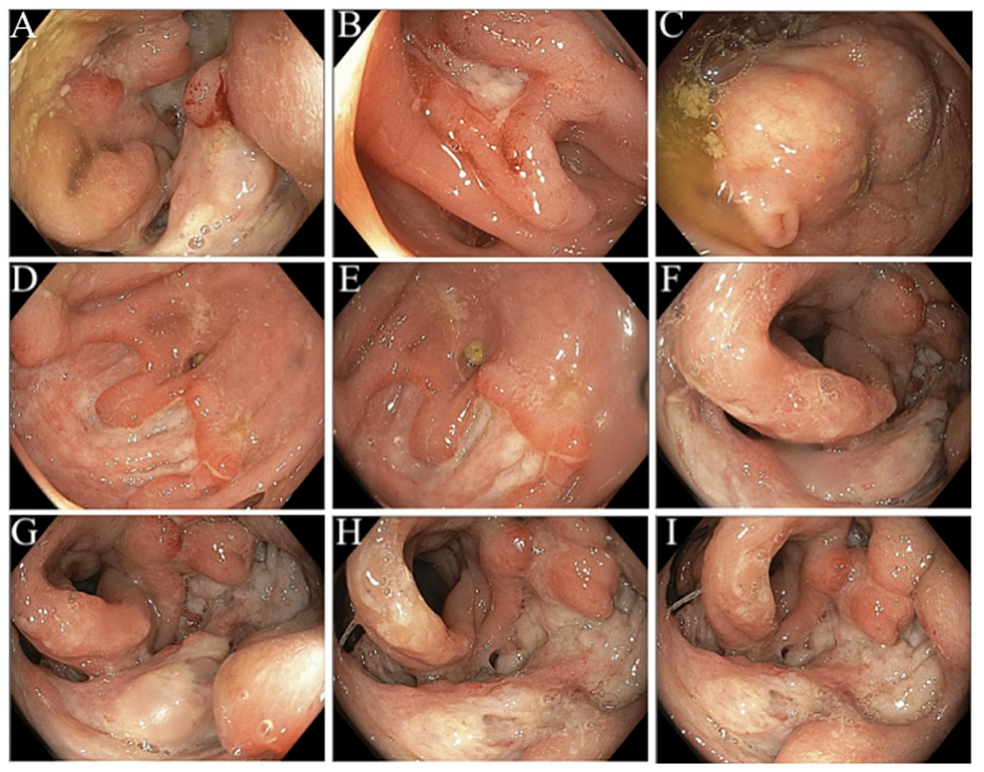
Images captured on colonoscopy

The patient’s colonoscopy was significant for severe luminal narrowing of the rectosigmoid colon with localized deep circumferential mucosal ulcerations, mucosal edema, erythema, and friability. The affected mucosa was hard to touch with the forceps. The lesion extended from 15 to 18 cm from the anal verge (Figures 1A-1I). Biopsies of the affected area showed ulceration and active colitis but malignancy could not be excluded.

**Figure 2:**
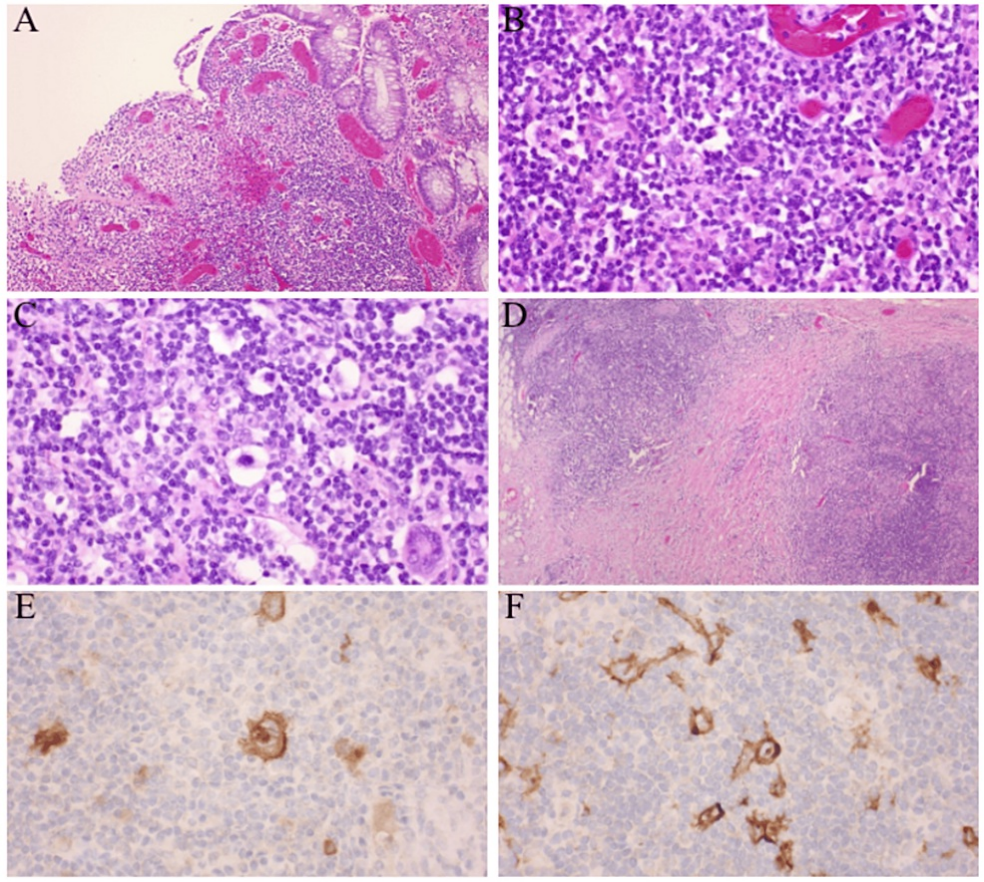
2a shows the ulcerated sigmoid colon mucosa with atypical infiltrate (100X; H&E stain); 2B, 2C show an atypical infiltrate including Reed-Sternberg cells (400X; H&E stain); 2D shows a pericolonic lymph node with nodular sclerosing Hodgkin’s disease (400X; H&E); 2E and 2F show CD30 immunohistochemistry stain highlighting Reed-Sternberg cells (400X; H&E) H&E: hematoxylin and eosin

Repeat laboratory findings following the procedure are detailed in Table 1. Following colonoscopy, CT abdomen and pelvis was significant for worsening moderate to severe distal proctocolitis with superimposed distal colonic diverticulosis. CT of the chest showed no findings suggestive of metastatic lesions. Carcinoembryonic antigen level was normal. Due to high suspicion of malignancy and the risk of colonic obstruction, the patient underwent a robotic-assisted lower anterior resection of the rectosigmoid mass and peri-colonic lymph node excision. Histology of the postoperative specimen showed an atypical lymphoid infiltrate characterized by a polymorphous proliferation of lymphocytes, plasma cells, rare eosinophils, scattered reed Sternberg cells, and atypical mononuclear and multinucleated Hodgkin’s variants (Figures 2A-2C). The majority of lymph nodes appeared to be reactive with prominent germinal center hyperplasia and some nodes showed partial involvement with the atypical lymphoid infiltrate along with several demonstrating early collagen bands, lacunar cells, and R-S cells, consistent with nodular sclerosing subtype (Figure 2D). Immunohistochemistry stains were positive for CD30 and CD15 (Figures 2A-2E).

Repeat laboratory data are provided in Table 1.

**Figure 3:**
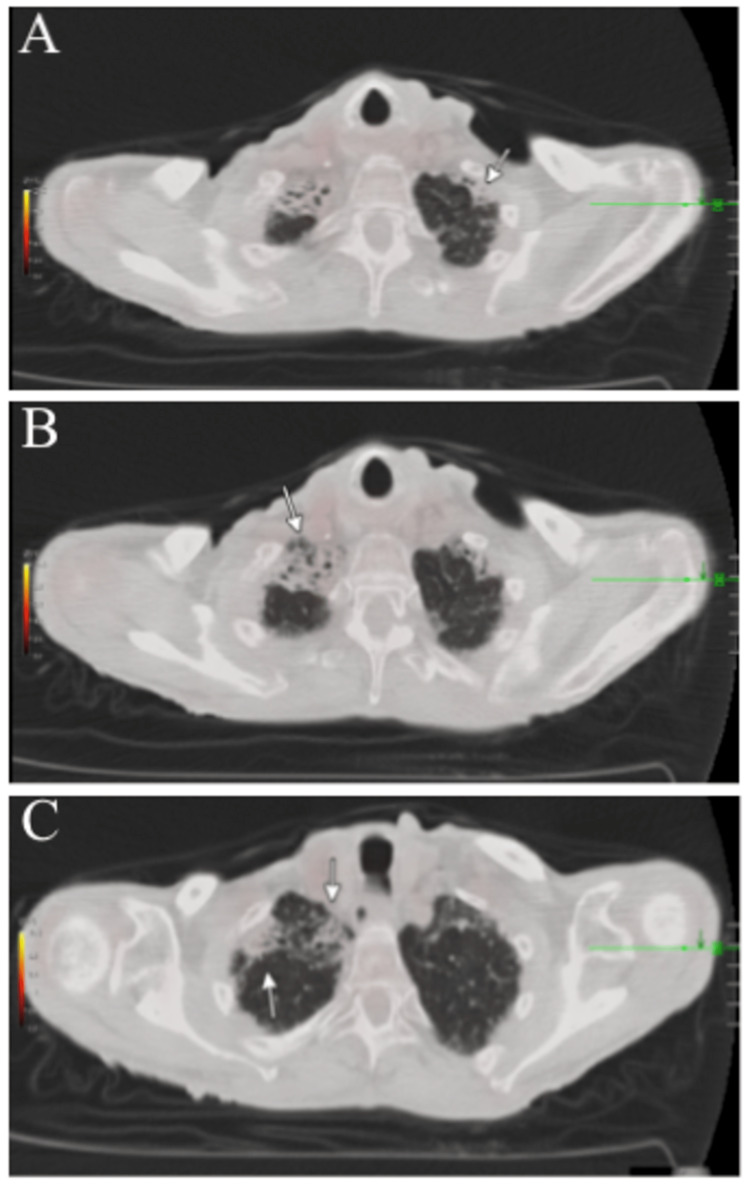
Positron emission tomography CT skull base to mid-thigh; coronal section demonstrating pulmonary fibrotic changes in the lungs CT: computed tomography

**Figure 4:**
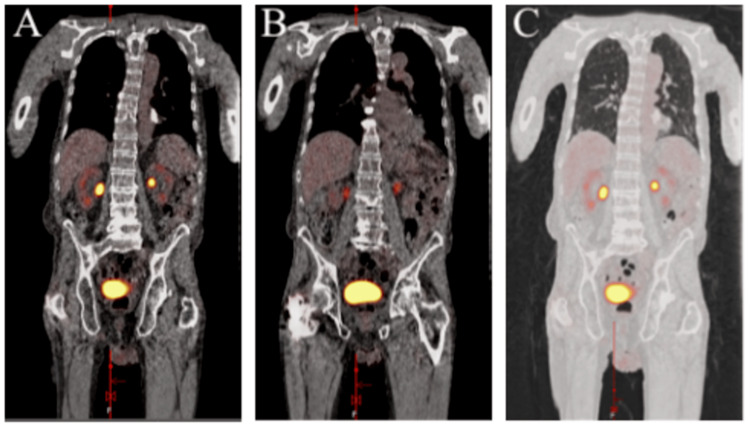
Positron emission tomography CT skull base to mid-thigh showing the absence of abnormal FDG avidity in the chest, abdomen, or pelvis. There are no solid lung lesions. CT: computed tomography; FDG: F-fluorodeoxyglucose

The positron emission tomography (PET) scan showed pulmonary interstitial and fibrotic changes (arrow in Figures 3A-3C coronal section) with no evidence of abnormal F-fluorodeoxyglucose (FDG) avidity in the chest, abdomen, or pelvis (Figures 4A-4C). The patient was referred to hematology and oncology, where options for chemotherapy/radiation versus monitoring were discussed, and the patient chose the latter. The patient's Eastern Cooperative Oncology Group (ECOG) performance status was one and the Lugano classification was stage IE. Despite the patient relocating, he maintained follow-ups with oncology every three months. The patient was found to have no recurrence of lymphoma; however, he passed away 15 months after the diagnosis due to undocumented reasons.

## Discussion

The gastrointestinal tract is the most common extranodal site of lymphoma involvement, making up 5-20% of all cases [5]. However, primary gastrointestinal lymphoma is very rare. Non-Hodgkin’s lymphoma makes up the majority of primary GI lymphomas, with Hodgkin’s lymphomas being in the minority. The most common site of primary gastrointestinal lymphoma is the stomach, followed by the small intestine, and then the colon [6,7]. Of those primary lymphomas of the colon, the most common sites of disease were the cecum (60%), followed by the ascending colon (27%), and then the sigmoid colon (13%) [8]. Typically, Hodgkin’s lymphoma of the colon appears as ulcers or strictures on colonoscopy. When combined with histology, the inflammatory background of Hodgkin’s lymphoma can resemble inflammatory bowel disease, which is rarely associated with Hodgkin’s lymphoma [9]. The presence of Reed-Sternberg cells on histology confirms the diagnosis of Hodgkin’s lymphoma.

The presented patient was a 79-year-old immunocompetent male with a history of irritable bowel syndrome with diarrhea (IBS-D) who presented with persistent diarrhea for six months associated with a 15-pound weight loss and was eventually diagnosed with primary Hodgkin’s lymphoma of the rectosigmoid colon.

Diagnosis of IBS relies on the Rome IV criteria, requiring recurrent abdominal pain for at least one day per week for at least three months and includes two of the following: pain related to defecation, pain corresponding to a change in frequency, or appearance of stool. IBS is a relapsing and remitting disorder and usually poses no cancer risk [10].

Primary colonic lymphomas, including Hodgkin’s lymphoma, typically present with non-specific symptoms in patients in older age groups, which can lead to advanced staging on presentation and possible delays in diagnosis. Treatment options include combination chemotherapy with ABVD (doxorubicin, bleomycin, vinblastine, and dacarbazine) for most patients; however, for select patients, an acceptable alternative regimen includes BV+AVD (brentuximab vedotin, doxorubicin, vinblastine, and dacarbazine) or BEACOPP (bleomycin, etoposide, doxorubicin, cyclophosphamide, vincristine, procarbazine, and prednisone). We share this case to bring attention to and raise awareness of colonic Hodgkin’s lymphoma as a rare cause of chronic diarrhea and weight loss. Even in a patient with a history of IBS-D, persistent symptoms not typical of IBS warrants further workup to avoid potential delay of care and adverse morbidity and mortality outcomes.

## Conclusions

Due to its rarity, there is a paucity of data regarding prognosis, survival, and response to therapy in patients with primary Hodgkin’s lymphoma of the GI tract. Most of the data are derived from case reports and case series. We recommend that primary care providers and gastroenterologists when presented with a patient with a history of IBS, B symptoms, including fever, night sweats, and weight loss, and/or symptoms of IBS that are unresponsive to traditional treatment options, consider this to be a potential diagnosis on their differential list. To improve patient outcomes, there is a need for more research in this area and the establishment of a patient registry could potentially help.
